# PROGRAM OF MERIT REVIEWS: THE REVIEWERS’ PERSPECTIVE

**DOI:** 10.1093/geroni/igz038.2116

**Published:** 2019-11-08

**Authors:** Donna Weinreich

**Affiliations:** Western Michigan University, Kalamazoo, Michigan, United States

## Abstract

Each Program of Merit Application goes through a process of review. It is the same regardless if the application is for a gerontology program or a health professions program. In this session, the reviewer perspective will be shared and tips for how to complete an application to provide information in a format that aids the review will be shared.

